# The Relationship between *AMH* and *AMHR2* Polymorphisms and the Follicular Phase in Late Reproductive Stage Women

**DOI:** 10.3390/ijerph13020185

**Published:** 2016-02-02

**Authors:** Anna Jurczak, Małgorzata Szkup, Anna Grzywacz, Krzysztof Safranow, Elżbieta Grochans

**Affiliations:** 1 Department of Nursing, Pomeranian Medical University in Szczecin, Żołnierska 48, 71-210 Szczecin, Poland; jurczaka@op.pl (A.J.); m.szkup@onet.eu (M.S.); 2 Department of Psychiatry, Pomeranian Medical University in Szczecin, Broniewskiego 26, 71-460 Szczecin, Poland; annagrzywacz@gazeta.pl; 3 Department of Biochemistry, Pomeranian Medical University in Szczecin, Powstańców Wielkopolskich 72, 70-111 Szczecin, Poland; chrissaf@mp.pl

**Keywords:** late reproductive stage, follicular phase, *AMH*, *AMHR2*

## Abstract

The objective of this work was the analysis of the relationships between the genotypes of the AMH and AMH receptor type 2 genes, hormone levels and the menstrual cycle in a group of Polish women *in the late reproductive stage.* The study was conducted using a measurement-based method (body weight and height), laboratory method (serum hormone levels AMH, FSH and E2), and genetic analysis (DNA isolated from whole blood by a salting-out method). The study involved 345 healthy, late-reproductive-stage women from Poland, aged 42.3 ± 4.5 years. The analysis demonstrated that neither the T/T and G/T+G/G genotypes of the *AMH* Ile^49^Ser polymorphism (rs10407022), nor the A/A and the G/A + G/G genotypes of the *AMHR2* 2482 A > G polymorphism (rs2002555), nor the C/C and C/T + T/T genotypes of the AMH polymorphism (rs11170547) were statistically significantly related (*p* > 0.05) to such factors as age, BMI, hormone (FSH and E_2_) levels and ovarian parameters (AMH) in the follicular phase. No relationships were found between ovarian parameters (FSH, E_2_, AMH) and genetic variants of *AMH* (rs10407022) and *AMHR2* (rs11170547, rs2002555) in healthy women in the late reproductive stage.

## 1. Introduction

Anti-Müllerian hormone (AMH) belongs to the transforming growth factor-β family. During folliculogenesis, AMH expression starts in the granulosa cells of the Graafian follicles. It is highest in the granulosa cells of preantral and small antral follicles, and then gradually decreases at the successive stages of the follicle development [1]. Mouse research suggests that the lack of AMH causes follicles to be recruited at a considerably faster pace. It has been also observed that follicles display increased sensitivity to follicle stimulating hormone (FSH) [2].

Studies of women mainly focus on the role that serum AMH plays as an indicator of ovarian function. The study of van Rooij *et al.* [3] demonstrated a strong relationship between serum AMH levels and the antral follicle count (AFC) assessed by means of an ultrasound scanner and reflecting the size of the primordial follicle reserve. On the basis of this study AMH was found to be an excellent marker of ovarian reserve.

The role of AMH in human ovarian physiology has not been sufficiently examined yet. Based on the pattern of AMH expression in women, it can be assumed that AMH influences the functioning of ovaries through *its* inhibitory effect on primordial follicle recruitment and FSH sensitivity [4]. Riggs *et al.* [5] observed that AMH levels correlated with the levels of ovarian reserve and the number of oocytes retrieved. This hormone has been recognized as highly predictive of fertility, after taking into account such factors as age and the levels of the following hormones: FSH, luteinizing hormone (LH) and estradiol (E2) [5].

Some of the studies conducted so far demonstrate that *AMH* polymorphism (rs10407022) located on the chromosome 19p13.3, and *AMHR2* polymorphisms (rs2002555 and rs11170547) located on the chromosome 12q13 are related to estradiol levels during the follicular phase in normo-ovulatory women. This relationship suggests that polymorphisms of these genes may influence the regulation of FSH sensitivity [6]. It is suspected that *AMH* polymorphism (rs10407022) and *AMHR2* polymorphisms (rs2002555 and rs11170547) may have significant effects on the biological activity of hormones involved in the control of the development and recruitment of follicles [7]. Genetic variants of *AMH* and *AMHR2* may have effects on hormone metabolism during folliculogenesis, and thus contribute to fertility [7]. The rs10407022 c.146T > G polymorphism leads to the amino acid substitution in the protein: isoleucine to serine in position 49 of the AMH protein (Ile49Ser). Since it is responsible for protein stability, mutations within this region could affect AMH biosynthesis or bioactivity, or even inactivate the protein [8].

Analysis of the polymorphisms of genes playing an important role in the regulation of female reproductive functions can improve understanding of mechanisms which influence the functioning of gonads and female fertility [9]. In the present study, the function of AMH in normally menstruating women was investigated. It was done by means of genetic analysis of the polymorphisms that may contribute to individual differences in the dynamics of the menstrual cycle through coding proteins involved in this process. The analysis was based on tagging of SNPs selected out of the genes involved in initial follicle recruitment, namely rs10407022 in *AMH* and rs2002555 and rs11170547 in *AMHR2*.

### Aim of the Study

Analysis of the relationships between the genotypes of the *AMH* and AMH receptor type II (*AMHR2*) genes, hormone levels and the menstrual cycle in a group of Polish women in the late reproductive stage.

## 2. Material and Methods

The study involved 345 healthy, late-reproductive-stage women from northwest Poland. The criteria for inclusion in the study were: no endocrine disorders, no gynecological disorders (the women had normal smear test results, and normal mammogram/breast ultrasound results), no neoplastic diseases, and no psychiatric problems. The criteria for exclusion from the study were: neoplasms of breast, neoplasms of the reproductive organs, endocrine disorders, abnormal smear test results, diagnosis of thyroid diseases and/or diabetes, diagnosis of neoplastic diseases, and diagnosis of mental diseases. Blood was collected from the participants on the third day of the menstrual cycle *i.e.*, in the early follicular phase. The study was conducted using the following methods:

### 2.1. Morphometry

The participants’ body weight and height were measured. Next their Body Mass Index (BMI) was calculated on the basis of the formula: weight in kilograms divided by height in meters squared (kg/m^2^).

### 2.2. Laboratory

The blood for laboratory analysis was collected using the BD Vacutainer blood collection system in accordance with the recommended guidelines [10]. The levels of AMH, FSH and E2 were determined in a certified laboratory (certificate no. ISO 9001:2008). For the follicular phase we accepted the norms of the “Medis” laboratory, namely: E2—12.5–166 pg/mL, FSH—3.5–12.5 mlU/mL [11]. We applied immunoassay for the *in vitro* quantitative determination of AMH in human serum, and an electrochemiluminescence method (ECLIA) for use in Elecsys (cobas e analyzers). Measurements were done using the sandwich method. Total duration of each assay was 18 min and consisted of a 1st incubation and a 2nd incubation. In the 1st incubation sample (50 µL), a biotinylated AMH-specific antibody, and a monoclonal AMH-specific antibody labeled with a ruthenium complex reacted to form an immunological sandwich complex. In the 2nd incubation: after addition of streptavidin-coated microparticles, the complex became bound to the solid phase via the interaction between biotin and streptavidin. The reaction mixture was aspirated into the measuring chamber where the microparticles were magnetically captured onto the surface of the electrode. Unbound substances were then removed with ProCell/ProCell M. Application of a voltage to the electrode then induced a chemiluminescent emission which was measured using a photomultiplier. Results were determined via a calibration curve which is instrument specifically generated by 2-point calibration and a master curve provided via the reagent barcode. The data were generated using Roche’s assay. The samples were measured on different days in accordance with the procedure guidelines. The limit of detection (LOD) is 0.010 ng/mL, (0.071 pmol/L), limit of quantitation (LOQ) is 0.030 ng/mL, (0.214 pmol/L) and limit of blank (LoB) is 0.007 ng/mL (0.05 pmol/L) [12]. On the basis of the results the women were categorized as being in the late reproductive stage. The next stage of the study was based on genetic analysis, in which DNA was isolated from whole blood by a salting-out method according to Miller *et al.* [13].

### 2.3. Genotyping

All laboratory procedures were carried out blind to diagnostic assessment. All genotyping was performed by fluorescence resonance energy transfer real-time PCR using the Light Cycler System 2.0. For the polymorphisms in the *AMH* and *AMHR2* genes the following conditions were applied: polymerase chain reaction (PCR) was performed with 50 ng DNA in a total volume of 20 mL containing 2 mL reaction mix, 0.5 mM of each primer, 0.2 mM of each hybridization probe and 2 mM MgCl_2_ according to the manufacturer’s instructions for 35 cycles of denaturation (95 °C for 10 min), annealing (60 °C for 10 s) and extension (72 °C for 15 s). After amplification, a melting curve was generated by holding the reaction at 40 °C for 20 s and then heating slowly to 85 °C. The fluorescence signal was plotted against temperature to give melting curves for each sample (Table 1).

**Table 1:** Analysis of melting curves for different alleles of *AMH* and *AMHR2*.

*AMH* (rs10407022)	*AMHR2* (rs2002555)	*AMHR2* (rs11170547)
For allele G Tm = 57.99 (°C)	For allele A Tm = 52.26 (°C)	For allele T Tm = 55.07 (°C)
For allele T Tm = 66.23 (°C)	For allele G Tm = 61.22 (°C)	For allele C Tm = 62.37 (°C)

(Tm)—melting temperature.

We identified polymorphisms in the *AMH* and *AMHR2* genes. Next, we analyzed the relationships between the polymorphisms of these genes and the levels of hormones and ovarian parameters in healthy women of the Caucasian race in the premenopausal period (the late reproductive stage according to the STRAW + 10 staging system). All participants gave their informed consent to participate in the research. The study was conducted in accordance with the Declaration of Helsinki, and the protocol was approved by the Bioethical Commission of the Pomeranian Medical University in Szczecin (permit number KB-0012/12/12).

### 2.4. Statistical Analyses

Statistical analysis was performed using Statistica 10 PL. The values of quantitative variables were presented as mean ± standard deviation (SD) or median (interquartile range (IQR) since the latter are better suited for non-normally distributed variables. The non-parametric Mann-Whitney U test was employed to compare variables between genotype groups. Multiple linear regression was used to analyze association of AMH (log-transformed due to right-skewed distribution) as dependent variable with genotypes and age as independent variables. The level of statistical significance was set at *p* ≤ 0.05.

## 3. Results

The women included in the study were aged 42.3 ± 4.5 (median 42 years). More than half of them (75.1%) had completed higher education, 22.6%—secondary education, 2.0%—vocational education, and 0.3%—primary education. The majority of the women lived in cities with a population of more than 100,000 residents (72.5%); 11.9% and 2.9% lived in rural areas and towns with a population of up to 10,000, respectively; the remainder (12.7%) lived in towns with less than 100,000 residents. The majority of the participants had life partners (75.1%). Nearly all women were employed (96.5%). Most women (61.0%) had been pregnant more than once, and 12.2% had never been pregnant. (Table 2) The mean ± SD and median (IQR) values of BMI, FSH, E2 and AMH were respectively: 24.73 ± 4.15, 24.2 (4.9) kg/m^2^; 13.41 ± 36.81, 6.4 (3.5) mIU/mL; 121.03 ± 111.97, 80 (96.1) pg/mL; 2.07 ± 2.17, 1.33 (2.34) ng/mL.

**Table 2:** Sociodemographic data.

Variables		*n* (%)
Marital status	single	47 (13.6)
	married	259 (75.1)
	divorced	37 (10.7)
	widow	2 (0.6)
Education	primary	1 (0.3)
	vocational	7 (2.0)
	secondary	78 (22.6)
	higher	259 (75.1)
Place of residence	village	41 (11.9)
	town of up to 10,000 residents	10 (2.9)
	town of up to 100,000 residents	44 (12.7)
	city with more than 100,000 residents	250 (72.5)
Employment status	employed	331 (96.5)
	unemployed	12 (3.5)
Number of pregnancies	0	42 (12.2)
	1	92 (26.8)
	>1	210 (61.0)

*n*—number of participants.

The genotype distributions of all three polymorphisms were perfectly consistent with Hardy-Weinberg equilibrium (*p* = 1 for each). The analysis demonstrated the lack of statistically significant associations (*p* > 0.05) between the genotypes of the *AMH* Ile^49^Ser polymorphism (rs10407022), *AMHR2* -482A > G (rs2002555) and C > T rs11170547 polymorphisms and such factors as age, BMI, hormone (FSH and E_2_) levels and ovarian parameters (AMH) during the follicular phase in healthy women in the late reproductive stage (Table 3, Table 4 and Table 5).

Since *AMH* concentration strongly negatively correlated with age (Pearson *r* = −0.45, *p* < 0.000001 for log-transformed *AMH* values) we performed multivariate analysis adjusted for age. Still no associations between *AMH* and the genotypes of rs10407022 (*p* = 0.99), rs2002555 (*p* = 0.32) and rs11170547 (*p* = 0.45) were found.

To verify the hypothesis that some genotype-phenotype associations might be present only in younger women and not in the whole study group, we stratified the women according to median age into younger (<42 years, *n* = 158) and older (≥42 years, *n* = 187) subgroups. However no significant association was found in any of the subgroups (data not shown). When multivariate analysis was performed, including age subgroup, genotype and interaction between them as independent variables, the older age remained a significant factor associated with lower *AMH* concentrations (β values between −0.40 and −0.45 for models with different polymorphisms, *p* < 0.000001), but neither associations with the genotypes (*p* > 0.3) nor with the interaction terms (*p* > 0.15) were found.

**Table 3:** The genotypes of the *AMH* Ile^49^Ser polymorphism (rs10407022) *vs.* age, BMI, hormone levels and ovarian parameters in the follicular phase in healthy late reproductive stage women (*n* = 345).

Variables	T/T *n* = 252 (73%)		G/T + G/G *n* = 93 (27%)		*p*
	**$\bar{x}$ ± SD**	**M (IQR)**	**$\bar{x}$ ± SD**	**M (IQR)**	
Age (years)	42.1 ± 4.5	42 (7)	42.9 ± 4.6	42 (6)	0.13
BMI (kg/m^2^)	24.7 ± 4.0	24.2 (4.6)	24.9 ± 4.5	24.1 (5.6)	0.99
AMH (ng/mL)	2.1 ± 2.2	1.4 (2.4)	1.9 ± 2.0	1.2 (2.3)	0.45
FSH (mIU/mL)	13.4 ± 40.8	6.3 (3.4)	13.4 ± 22.8	6.5 (4.2)	0.98
E_2_ (pg/mL)	120.9 ± 113.2	78.7 (94.8)	121.4 ± 109.2	84.5 (104.1)	1.00

*n*—number of participants, $\bar{x}$—arithmetic mean, SD—standard deviation, M—median, IQR—interquartile range, *p*—level of significance.

**Table 4:** The genotypes of the *AMHR2* 2482 A > G polymorphism (rs2002555) *vs.* age, BMI, hormone levels and ovarian parameters in the follicular phase in healthy late reproductive stage women (*n* = 345).

Variables	A/A *n* = 248 (71.9%)		G/A + G/G *n* = 97 (28.1%)		*p*
	**$\bar{x}$ ± SD**	**M (IQR)**	**$\bar{x}$ ± SD**	**M (IQR)**	
Age (years)	42.3 ± 4.6	42 (6)	42.4 ± 4.4	42 (7)	0.68
BMI (kg/m^2^)	24.6 ± 3.9	24.2 (4.8)	25.0 ± 4.8	24.2 (5)	0.97
AMH (ng/mL)	2.2 ± 2.3	1.3 (2.5)	1.8 ± 1.8	1.4 (2.2)	0.42
FSH (mIU/mL)	11.9 ± 19.9	6.2 (3.6)	17.3 ± 61.8	6.8 (3)	0.17
E_2_ (pg/mL)	119.7 ± 104.7	82.8 (94.9)	124.5 ± 129.3	77.2 (102.4)	0.98

*n*—number of participants, $\bar{x}$—arithmetic mean, SD—standard deviation, M—median, IQR—interquartile range, *p*—level of significance.

**Table 5:** The genotypes of the *AMHR2* polymorphism (rs11170547) *vs.* age, BMI, hormone levels and ovarian parameters in the follicular phase in healthy late reproductive stage women (*n* = 345).

Variables	C/C *n* = 279 (80.9%)		C/T + T/T *n* = 66 (19.1%)		*p*
	**$\bar{x}$ ± SD**	**M (IQR)**	**$\bar{x}$ ± SD**	**M (IQR**	
Age (years)	42.2 ± 4.6	42 (6)	42.6 ± 4.4	42 (8)	0.49
BMI (kg/m^2^)	24.7 ± 4.2	24 (4.9)	24.8 ± 3.8	24.4 (5.1)	0.59
AMH (ng/mL)	2.1 ± 2.2	1.4 (2.5)	1.8 ± 1.9	1.3 (2.1)	0.31
FSH (mIU/mL)	14.4 ± 40.8	6.2 (3.6)	9.1 ± 6.5	7.1 (3.6)	0.07
E_2_ (pg/mL)	120.5 ± 105.3	81.4 (94.9)	123.2 ± 137.6	75.7 (105.3)	0.69

*n*—number of participants, $\bar{x}$—arithmetic mean, SD—standard deviation, M—median, IQR—interquartile range, *p*—level of significance.

## 4. Discussion

The study conducted by Kevenaar *et al.* [6] among normo-ovulatory women in Dutch and German cohorts shows that the rs10407022 and rs2002555 polymorphisms have significant influence on the level of estradiol during the early follicular phase of the menstrual cycle. These authors observed significantly higher estradiol levels on the third day of the menstrual cycle in Dutch women with the T/T genotype than in other participants of the study. When analyzing the Dutch and German cohorts together, the authors repeated their observation, suggesting that carriers of the T/T genotype have higher estradiol levels. However, the relationships between the above mentioned polymorphisms and the levels of AMH and FSH in serum were not confirmed [6].

Other results indicate that AMH may regulate estradiol levels through its ability to modulate ovarian sensitivity to FSH [14]. It has been noticed that women who are the carriers of the T allele of the *AMH* gene or the G allele of the *AMHR2* gene and at the same time have increased estradiol levels may have lower FSH threshold levels. It results in more effective stimulation of estradiol production during the follicular phase. This relationship is even more evident in those who are the carriers of both the T allele of *AMH* and the G allele of *AMHR2* [8]. In the study of the German cohort, carriers of the G allele of *AMHR2* (rs2002555) had higher estradiol levels in the early follicular phase, than their counterparts who did not have this allele. The results show that the G allele predisposes to higher sensitivity to FSH, which in turn may lead to the shortening of the follicular phase [15].

Our study did not confirm significant differences between estradiol levels depending on the presence of specific rs10407022 and rs2002555 alleles. It is important, however, to take into consideration characteristics of the group ‒ it included women in the late reproductive stage (aged 42.3 ± 4.5, median 42 years). During the perimenopausal period (35+ years), estrogen levels rise in an unpredictable manner, due to disturbed feedback mechanisms. Kevenaar *et al.* [6] obtained different results, probably because they analyzed women at the age of 20–35 years. Peluso *et al.* [7] did not observe the relationship between estradiol levels and the presence of rs10407022 in AMH and rs2002555 in AMHR2. A study of Chinese infertile women also did not demonstrate correlations between serum estradiol levels and the rs10407022 polymorphism [16]. The results obtained by other authors also did not provide evidence that FSH levels are related to the *AMH* and the *AMHR2* genes. The only significant relationship observed by Peluso *et al.* [7] was the one between the rs3741664 polymorphism (not analyzed in our study) and the FSH level. The results of our study suggest that the *AMH* C/T and/or T/T genotypes (rs11170547) might be advantageous with respect to reproduction because the mean FSH level in this group is lower (9.1 ± 6.5) than in any of the other genotypes (14.4 ± 40.8). This difference is almost at the level of significance (*p* = 0.07).

The T/T genotype of the rs10407022 polymorphism reduces sensitivity of antral follicles to FSH less effectively than the G/G genotype. In vitro analyses conducted by Kevenaar *et al.* [8] demonstrated that carriers of the T/T genotype were characterized by lower protein bioactivity than individuals with the G/G genotype. It resulted in the decline in a total number of antral follicles among these individuals comparing to other groups. These results were not confirmed by studies of other authors [7]. 

As a limitation of our study, we investigated women in the late reproductive stage. The current data set shows that *AMH* polymorphisms are not related to estrogen levels. In the investigated women, aged 42.3 ± 4.5 (median 42 years), the normal regulatory control of estrogen had begun to breakdown. Estrogen levels are dysregulated in older women, and their data is therefore uninformative of whether *AMH* polymorphisms affect estrogen levels in younger women. Further research is necessary to compare the results obtained for late reproductive stage women with those for normovulatory and perimenopausal groups.

## 5. Conclusions

No relationships were found between ovarian parameters (FSH, E2, AMH) and genetic variants of *AMH* (rs10407022) and *AMHR2* (rs11170547, rs2002555) in healthy women in the late reproductive stage. 
